# Valuation of Green Walls and Green Roofs as Soundscape Measures: Including Monetised Amenity Values Together with Noise-attenuation Values in a Cost-benefit Analysis of a Green Wall Affecting Courtyards

**DOI:** 10.3390/ijerph9113770

**Published:** 2012-10-24

**Authors:** Knut Veisten, Yuliya Smyrnova, Ronny Klæboe, Maarten Hornikx, Marjan Mosslemi, Jian Kang

**Affiliations:** 1 Institute of Transport Economics (TOI), Gaustadalleen 21, NO-0349 Oslo, Norway; Email: rk@toi.no (R.K.); marjan.mosslemi@sweco.no (M.M.); 2 School of Architecture, University of Sheffield, Western Bank, Sheffield S10 2TN, UK; Email: y.smyrnova@sheffield.ac.uk (Y.S.); j.kang@sheffield.ac.uk (J.K.); 3 Building Physics and Services, Eindhoven University of Technology, P.O. Box 513, 5600 MB Eindhoven, The Netherlands; Email: m.c.j.hornikx@tue.nl

**Keywords:** hedonic pricing, noise control, quiet area, quiet side, unit pricing, vertical gardens

## Abstract

Economic unit values of soundscape/acoustic effects have been based on changes in the number of annoyed persons or on decibel changes. The normal procedure has been the application of these unit values to noise-attenuation measures affecting the noisier façade of a dwelling. Novel modular vegetation-based soundscape measures, so-called green walls, might be relevant for both noisy and quieter areas. Moreover, their benefits will comprise noise attenuation as well as non-acoustic amenity effects. One challenge is to integrate the results of some decades of non-acoustic research on the amenity value of urban greenery into design of the urban sound environment, and incorporate these non-acoustic properties in the overall economic assessment of noise control and overall sound environment improvement measures. Monetised unit values for green walls have been included in two alternative cases, or demonstration projects, of covering the entrances to blocks of flats with a green wall. Since these measures improve the noise environment on the quiet side of the dwellings and courtyards, not the most exposed façade, adjustment factors to the nominal quiet side decibel reductions to arrive at an estimate of the equivalent overall acoustic improvement have been applied. A cost-benefit analysis of the green wall case indicates that this measure is economically promising, when valuing the noise attenuation in the quieter area and adding the amenity/aesthetic value of the green wall.

## 1. Introduction

Recent efforts of researchers, planners, architects, entrepreneurs and authorities dealing with increasing noise problems in European cities and urban areas have put an increased emphasis on the quality of soundscape [1,2,3]. Noise is understood as a sound that is loud, unpleasant, unexpected, or undesired, while soundscape can be defined as a sound or combination of sounds that forms or arises from an immersive environment; referring to both the natural acoustic environment and sounds generated from human activity [2]. These aspects have also received special attention through the protection of quiet areas in The Environmental Noise Directive (2002/49/EC). As a result there is also an increased focus on vegetation-based noise abatement measures, such as shrubs, trees and bushes, green barriers, and green façades and roofs that absorb, scatter, and affect the reflection of sound [4,5,6]. Green roofs and green walls provide an environment that both reduces noise and provides opportunities for amplifying natural and artificial sounds and creating supportive, exciting, higher quality urban micro and macro spaces [7]. “Beautification is therefore not only a visual matter, but a multisensory. Neither is the acoustic aesthetics only a matter of noise protection, but it encompasses a soundscape entirety” [8].

Economic unit values of soundscape/acoustic effects have been based on changes in the number of annoyed persons or on decibel changes [9]. The normal procedure has been the application of these unit values to noise-attenuation measures affecting the noisier façade of a dwelling. However, novel vegetation-based soundscape measures might be relevant for both noisy and quieter areas [10]. Recently, adjustment (correction) factors for calculating the overall benefit with respect to sleep disturbances and annoyance of noise attenuation for quieter façades have been calculated and proposed [3,11,12]. In the current paper this has been taken into account in an application of green walls to blocks of flats, a demonstration project where the green walls primarily will affect sound propagation in an inner courtyard (the design of these green wall demonstration projects was part of the EU project “Holistic and sustainable abatement of noise by optimized combinations of natural and artificial means” [10], rather than the flat façades facing a busy street. Both the acoustic and aesthetic benefits of these demonstration projects in a cost-benefit analysis of this green wall case are assessed. A sensitivity analysis, allowing for uncertainty in inputs, is included within the economic assessment of the green wall demonstration projects.

Green walls yield additional aesthetic/amenity qualities compared to traditional noise-attenuation measures, like noise barriers of wood or glass. The monetary value of noise and aesthetic/amenity qualities have been estimated in separate fields of research, although in both cases the value estimates have been based on hedonic pricing of dwellings, showing the percentage effect of e.g. urban greenery on sale prices [13]. However, while valuation of noise changes directly or indirectly can be related to decibel changes, and thus be given a unit price, e.g., EUR/dB, that is applicable to noise measures in general [14,15,16], there is no readily applicable unit price for the aesthetic/amenity value of urban greenery. The aesthetic/amenity valuation has been only case-based. Based on a review of green roof/wall hedonic valuations studies, this paper takes these a step further and estimates unit values of green roofs/walls. These unit values will be weighted averages of estimates from reviewed studies, given as a Euro price per square meter green roof/wall, an annual valuation per affected person or household. Although acknowledging the theoretical objections to unit pricing of greenery, this paper aims to propose a practitioner approach proposing some initial unit values that can be tested in simplified cost-benefit analyses.

## 2. Theoretical Underpinnings and Literature Survey

### 2.1. Unit Values for Soundscape/Noise Changes

The current economic valuation approach to soundscape/noise changes is either a valuation per person change from annoyed to not annoyed (or vice versa) or per dB(A) change per person (or household/dwelling) per year [9]. As a noise indicator the EU standard evening and night-time weighted equivalent noise level on the most exposed façade is used [17]. Thus, for an improvement in sound quality one might consider an EU average of 20 EUR per dB per person per year [18], or (assuming a household size of approximately two persons) 40 EUR per dB per household per year. Normally this type of valuation is applied to the noisier façade, not the quiet façade in the inner courtyard as is the case for our demonstration projects. Valuation experiments assessing human reactions to their sound environment predominantly target noise annoyance focussing on situations with high levels of transportation noise [9,19]. The benefits of quieter areas and areas with improved sound quality, such as of restoration, restitution from physical and mental illness, stress relief, positive experiences, physical activity *etc.* on health are not part of the valuation experiments. They are potentially important, but difficult to capture without appropriate complementary research approaches using other types of acoustic measures to assess the quality of the sound environment [20]. A consequence is that current valuations are likely to underestimate the impacts from an adverse soundscape and fail to include benefits of good quality sound environments [21,22]. 

An overview of the benefits of adopting a broader approach including contextual factors is provided by Lercher [23]. Important nuances and differences in the overall exposure situation that are not reflected in the L_den_ value of the most exposed façade should be taken into account. A challenge when valuing an improved soundscape at lower noise levels is that current approaches employ a high cut-off value. Improvements below the cut-off value are given zero economic value. As an example, all noise reductions below 50 dB(A) are regarded as providing no economic value [19]. The rationale for using a cut off is that current valuation studies fail to detect significant differences in annoyance at lower noise levels. However, the lack of sensitivity to differences in human reactions may also be a result of the over reliance on a single indicator of the sound environment. De Kluizenaar *et al.*, Öhrström *et al.*, and Gidlöf-Gunnarsson *et al.* found significant health, psychological and physiological benefits in asses to indoor and outdoor quiet sides [3,24,25]. Amundsen *et al.* found a shift in exposure annoyance relationships from having the bedroom at a quiet side to be equivalent to a 5dB noise reduction on the most exposed side [11]. See Benfield *et al.* for a study of the human-activity noise impact on the perceived soundscape quality in protected areas [26]. In the CityHush project correction factors have been proposed to capture the impact of façade insulation, access to a quiet side, and an improved or noisy neighbourhood soundscape [12,27], and a cut-off value of 45 dB(A) was employed, and assessed the improvement in the noise level on the quiet side to be 20% of that associated with a corresponding noise level reduction on the most exposed façade. Thus, a 10 dB reduction in the noise level difference between most and least exposed side is assessed to have the same effect on human reactions as that of a 2 dB noise reduction on the most exposed side.

### 2.2. Vegetation-Based Noise Attenuation Measures

Generally, in terms of acoustic benefit, vegetation affects the sound field in urban environments through three mechanisms: sound absorption and sound diffusion, which occur when a sound wave impinges on the vegetation and is then reflected back; and sound level reduction, when a sound wave is transmitted through the vegetation [4,5,6,7]. Consequently, when vegetation is used on building façades the effectiveness of façade absorption can be greatly enhanced since there are multiple reflections. In build-up areas the absorption and diffusion effects are also useful for reducing the negative effect of reflections from the ground that often occur in outdoor sound propagation [7,28,29,30]. Additionally, green roofs, particularly when installed on non-flat roofs, cause noise reduction of diffracted waves that intrude a street without traffic or a courtyard [5]. For detailed description of impacts from green roofs and green walls, on locale climate, water management, energy use, *etc.*, as well as particular challenges, see e.g. [31,32,33,34,35].

### 2.3. Non-Acoustic Amenity Values of Green Roofs/Walls

The reviewed green roof/wall valuation studies are based on the use of the hedonic pricing method, where it is estimated how much dwelling characteristics impact on the property price or the rent [36]. With data on sale prices of dwellings and the dwelling characteristics, e.g., location, living space, condition, number of bathrooms, *etc.* (plus environmental characteristics), the dwelling prices are regressed on the set of characteristics, finding the percentage impact of the characteristic on the selling prices. The hedonic pricing studies on green roof/wall valuation do not mention noise/soundscape among the amenity benefits; they all stress the aesthetics (visual benefits) and mention either thermal or climate effects. Thus, although the noise/soundscape impact as part of the amenity value still cannot be ruled out completely, we may assume for simplicity that these value estimates omit noise/soundscape effects. Based on the country/city of the study, the year of the data collection, and the currency of the country, the original value estimates has been converted to Euro, and then simply updated to Euro 2010 values by the consumer price index (CPI). For property values, or the property value shares indicated from the studies, we applied a discount rate of 5% and a 50 year lifetime to calculate annuity values that are comparable to annual rents [37,38]. The unit price estimate is simply the Euro 2010 value divided by the area of the measure, yielding a crude square metre value of the greenery. We relate this valuation to a household, that is, what a household will pay per year for an additional square metre of greenery. The valuations can easily be converted to Euros per person by dividing by the average number of people living in a household; the average number of people in each household in the 27 Member States of the European Union was 2.4 in 2008 [39].

Peck *et al.* described both green roofs and green walls, assessing the market possibilities in Toronto (Canada) [34]. They assumed that these vegetation measures would yield the same property increase as “good tree cover”; and they stated a value increase interval for a property of 6–15%, thus a midpoint of 10.5%. For a Toronto house price of approximately CAD 230,000 in 1999, we calculated an annualised square metre value of green roofs and walls of approximately EUR 20 (2010 Euro value). This is the value accrued to one dwelling or household, not including possible external benefits for neighbours. Hunt does not differentiate between green walls and green roofs either, but states that: “The costs green upgrades add to a new home’s price vary widely. However, a 3 % to 15 % premium is a good rule of thumb” ([40] p. 1). This interval has a midpoint of 9%, which would yield approximately EUR 18, applying the same residential values as for Peck *et al.* [34]. Des Rosiers *et al.* estimated that hedges, or green walls, added 3.9% to the property value, in their hedonic price study applying property data from the city of Québec (Canada) [41].

Gao and Asami applied a particular kind of hedonic pricing of greenery, *i.e.*, greenery of walls as well as greenery of streets and pedestrian spaces [42]. They made estate agents value a set of dwellings in Tokyo and in Kitakyushu. The valuations were based on information about the sale prospects. Greenery quality scores were provided in addition to the street/landscape architecture attributes. For the aesthetic properties of walls they described three attribute levels: −1 for mostly concrete block walls, 0 for averagely greened walls, and +1 for continuously greened walls. Based on the pricing from the estate agents, they found that an increase in greenery quality level would increase land price by 1.4% in Tokyo and by 2.7% in Kitakyushu. If we assume 25 m^2^ as a green wall quantity approximation to (each of) the two quality levels (25 m^2^ greenery for level 0 and an additional 25 m^2^ greenery for level +1), the resulted unit value estimates is of, respectively, ca EUR 3.5 per square metre green wall in Tokyo and ca EUR 1 in Kitakyushu. Obviously, these estimates are very sensitive with respect to the area assumption.

Ichihara and Cohen found that homes in New York with green roofs had as much as 16.2% higher price than homes without green roofs [43]. Relative to an annual rental price of USD 4,000, a unit value per square metre green roof of ca EUR 17 can be estimated. In this case the homes were apartments in three 24–27 floor buildings with 252–279 units, such that the aggregate value would be huge compared to the value for one household. Tomalty and Komorowski present value estimates for two types of green roof, a recreational rooftop garden and a “productive” rooftop garden (including vegetables/fruit) [44]. For recreational green roofs they found a 20% property price increase, while for productive rooftop gardens they indicated a 7% increase. This study is from Toronto, but their estimates are also based on studies from other areas. It is possible to calculate that unit value estimates will be of, respectively, ca EUR 63 per square metre recreational rooftop garden and ca EUR 22 per square metre productive rooftop garden. Table 1 summarises the estimations for green roofs/walls.

**Table 1:** Value estimates, per household per year, green roof/wall ^†^.

Study	Year Data	Country Data	Curr-ency	Property Price	Annu-ity	Gree-nery % Value	Gree-nery Value	EUR/ Curr-ency	CPI	EUR-2010 Gree-nery Value	Gree-nery Size (m^2^)	Unit Value (per m^2^)
Peck *et al.* (1999) [34]	1999	Canada, Toronto	CAD	230,000	12,599	10.50%	1 323	0.6313	1.25	1,044	50	20.88
Hunt (2008) [40]	1999	Canada, Toronto	CAD	230,000	12,599	9%	1 134	0.6313	1.25	895	50	17.9
Gao & Asami (2007) [42]	1999	Japan, Tokyo	JPY	602,400	32,998	1.40%	8 400	0.0082	1.25	87	25	3.46
	2003	Japan, Kitakyushu	JPY	73,200	4,010	2.70%	1 980	0.0076	1.15	17	25	0.69
Ichihara *et al.* (2010) [43]	2000	US, New York	USD	73,024	4,000	16.20%	648	1.0827	1.22	859	50	17.18
Des Rosiers *et al.* (2002) [41]	1999	Canada, Québec	CAD	112,000	6,135	3.90%	239	0.6313	1.25	189	50	3.78
Tomalty & Komorowski (2010) [44]	2010	Canada, Toronto	CAD	395,460	21,662	20%	4 332	0.7325	1	3,174	50	63.47
	2010	Canada, Toronto	CAD	395,460	21,662	7%	1 516	0.7325	1	1,111	50	22.22

^†^ Values in currency of a study are converted to Euro in the year of the study, and these Euro values are adjusted to Euro 2010 values applying the consumer price index (CPI) for the Euro zone. A discount rate of 5% and a 50 year lifetime to calculate annuity values from the property values have been applied. If the study reports annual rental values, these are put under the annuity column. The “value” is the total green roof/wall annual value for the household. Since Hunt [40] reported only a percent interval of value effects, in this paper the same Toronto property values as for Peck *et al.* [34] has been applied. Thus, the EUR-2010 value equals the annuity times the greenery percentage value, times EUR/currency, times CPI; and the unit value equals the EUR-2010 value divided by the greenery size (in the valuation study).

The mean of these value estimates is EUR 18.7 per square metre greenery per household per year, but the mean drops to EUR 12.3 if the highest estimate from Tomalty and Komorowski is removed. This would yield approximately 5.1 EUR per affected person (assuming a household size of approximately 2.4 persons). A 90% confidence interval from the household unit value distribution is from about EUR 3 to about EUR 60. Due to the relatively low number of observations these estimated average unit values are statistically uncertain and unstable. The estimates also depend on the assumptions regarding the size of the valued object, as well as other quality aspects that has not been possible to control for [34,44,45].

## 3. Methodological Approaches to the Delineation of Unit Values

### 3.1. Unit Values for Soundscape/Noise Changes

This paper assesses cases where vegetation-based noise attenuation measures affect the quieter façades of dwellings and the common residential areas, the inner courtyards. The estimated Miedema curves for annoyance have 37 dB(A) and 42 dB(A) as departure values for the proportion of people who report they are annoyed or highly annoyed respectively [17]. A pragmatic approach is here to employ a cut-off value of 45 dB(A) as used by the CityHush project [12,27]. A conversion factor of 30% where 3 dB reduction in the sound level on the quiet side counts as 1 dB reduction on the most exposed side has been also chosen (somewhat higher than the 20% that was suggested in the CityHush project). This is motivated by the additional improvement of the courtyard soundscapes and the somewhat wider neighbourhood soundscape impacts of a project consisting of repeat interventions.

### 3.2. Validation of Non-Acoustic Amenity Unit Value Estimates

Meta-analyses are often used for synthesising results from various studies [46,47,48]. A first step in a simple meta-analysis of unit values is to estimate a weighted average, which takes into account differences in (reported) uncertainty, such that less uncertain estimates are given a larger weight. A weighted average can be estimated, where the weight of each study is based on the variance of the point estimate, the “effect”. In the case of this paper unit values (“effects”), *Y_g_*, where the subscript refers to study *g* (*g* = 1,...,*G*) has been extracted; *W_g_* is the statistical weight of the unit value estimate from study *g*, given from *W_g_* = *1/V_g_*, where *V_g_* = *SE*^2^*_g_* is the square of the standard error of the estimate. These are the optimal weights that minimise the variance of the combined estimate [49]. It should be noted though that the weights are determined by the intrinsic attributes of the survey. The weighting scheme does not take into account sources of variance due to differences in the type of valuation object, in cultural aspects, and in the design and execution of the valuation studies, *etc.* The reported precision might therefore overestimate the real precision of the estimates. It is also important to bear in mind the heroic assumptions behind the greenery unit value calculations, e.g., the implicitly assumed (perfect) divisibility of these greenery measures and the linearity of the value function. The value of a second square metre of urban greenery can be different from the value the first square metre; additional amounts of a good is often valued less due to differences in the general economic feature of diminishing marginal utility, which means that the value function will not be strictly linear. As long as there is a positive unit value, total value will increase though. No systematic research on how the valuation of roofs and façades vary with the amount/coverage of greenery has been found. In addition to the decreasing value of an extra unit for additional amounts of a given good, research on the spatial relationships between the good and the property it enhances show that the unit value decreases with the distance to the property. Tomalty and Komorowski present simple formulas for calculating expected property value increases from green roofs and other urban vegetation [44]. The main formula can simply be stated as: urban greenery value equals percentage impact on property/rental value, times property/rental value, times distance category.

Some referred studies have not reported measures of uncertainty in the price effect of urban greenery. Some do report price effect intervals, while others report the standard error of the price effect parameter in the hedonic regression model. In the latter case an underlying normal distribution of the parameter and estimate variance as the square of the estimated standard error have been assumed. When upper and lower levels are reported, it is assumed that these represent the upper and lower values of 95% confidence intervals, and, by assuming an underlying normal distribution, the implicit standard error from dividing by 1.96 on each side of the mean is extracted. When measures of uncertainty are missing, it is assumed that the standard error is 50% of the mean estimate. Moreover, it is assume that these uncertainty estimates can be applied to the EUR-2010 unit value point estimate.

## 4. Economic Assessment of a Green Wall Case

### 4.1. The Green Wall Demonstration Projects

**Figure 1:**
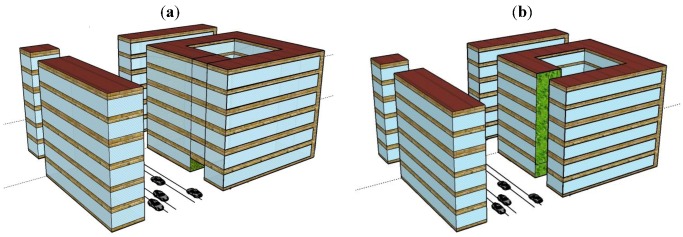
(**a**) Green walls at the entrance to an inner courtyard covering 58 m^2^, on a block of flats, six storeys high, with eight apartments on each floor; (**b**) Green walls at the entrance to an inner courtyard covering 369 m^2^ facade, on a block of flats, six storeys high, with eight apartments on each floor (Source: [51]).

To get some idea on how the estimated unit values of urban greenery might impact on estimated benefits of noise-control measures with aesthetical/ambient qualities, a test applied to two green wall applications has been included. These are based on two configurations, typical for European urban settings. They consist of a block of flats, six storeys high, with eight apartments on each floor. Both configurations should be compared to reference configurations without a green wall. The reference configurations present a part of an urban setting that consists of an urban street canyon with cross streets and a roadside courtyard. The main street canyon dimensions are 48 m × 19.2 m × 19.2 m (length × width × height), the cross street dimensions are 66.8 m × 9.6 m × 19.2 m and the courtyards dimensions 19.2 m × 19.2 m × 19.2 m. Due to periodicity in the computational model, the main street canyon is infinitely long, *i.e.* the configurations as shown in Figure 1(a,b) represent a single section of the actually modelled configuration. Road traffic with a flow of 20,000 vehicles/day (annual average daily traffic) and a speed of 50 km/h is present in the main street canyon, and is composed of 95% light and 5% heavy vehicles. No traffic is present in the cross street. The façades of the two reference configurations are equal and consist of windows and brickwork parts. Roadside courtyards are not always completely closed, *i.e.*, façade openings for convenient accessibility to the courtyard may be encountered. Therefore, a façade opening of 3 m × 9.6 m (height × width), in the first configuration and an opening of 19.6 m × 9.6 m (the opening runs through the whole height of the building) in the second configuration, both facing the street canyon, are considered (Figure 1(a,b)). Accordingly, a green wall will be placed at the walls of the entrance to an inner courtyard, either up to three metres or covering the full building height up to 19.2 m. Both sides of the opening are covered by vegetation of, respectively, 58 m^2^ (Figure 1(a)) and 369 m^2^ (Figure 1(b)). The green wall typically consists of a stainless steel container, geotextiles, a growing medium (substrate) with irrigation system and vegetation. The absorption of green wall drastically depends on the water content [50], and this study is performed for dry conditions, returning the maximum effects that can be obtained.

**Figure 2:**
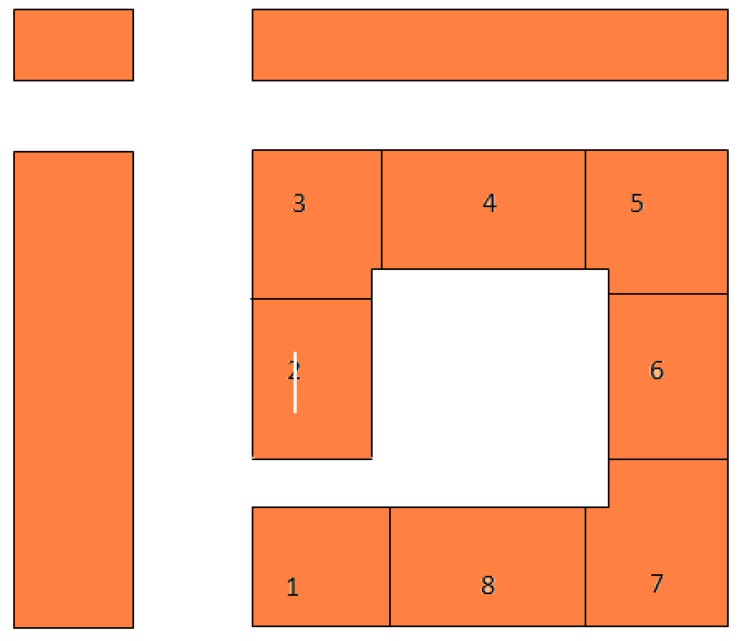
Top view of the one of the periods of the model representing the layout of the apartments at each floor.

All 48 apartments face both sides of the building, so 18 (3 × 6) apartments face a busy street as well as the courtyard, while 18 (3 × 6) apartments face the opposite side of the building and the courtyard, as can be seen from Figure 2. This leaves 12 (2 × 6) apartments facing the more quiet side streets and the courtyard. One of these sides, including one or both of the corner flats, that is two or three apartments at each floor, will in addition to the aesthetic improvement when using the immediate courtyard area, also have a direct view to the 19.2 m high green wall at the entrance façade (Figure 1(b)). For the alternative with only 3 m high green wall, it can be assumed that only two or three apartments at the ground floor will have a view to the green wall at the entrance façade (Figure 1(a)).

With the assumed road traffic as sources of sound, the sound pressure levels are computed by a hybrid approach. For the low frequency range, *i.e.*, up to 500 Hz, the PSTD method is. This is a numerical method solving the wave equation [52,53]. It captures all wave phenomena as relevant for these low frequencies and the configurations of Figure 1(a,b). For higher frequency range, *i.e.*, up to 4,000 Hz, the CRR energy-based method is used [54]. In this numerical method sound waves are treated as rays obeying rules of geometrical optics, thus no wave phenomenon is taken into account making it applicable for higher frequencies simulations. The 18 apartments (with about 30 persons) facing the street are exposed to a noise level of 75.7 dB(A), while the remaining 30 apartments (with about 70 persons) are exposed to a noise level of 56.3 dB(A) in the case of the small entrance (Figure 1(a)) and 59.3 dB(A) in the case of the large entrance (Figure 1(b)). The level in the courtyard is averaged over all receiver positions. The apartments facing the cross streets will have one façade with levels of 73.5 dB(A). The 19.2 m high green wall at the entrance façade will reduce the noise level by 4.1 dB for the quiet side of all 48 apartments, while the 3 m high green wall at the entrance façade will reduce the noise level by 4.5 dB for the quiet side of all 48 apartments. Finally, the apartments in the opposite building, without green wall, will have no noise attenuation impact from the green wall, whether it is three or 19.2 m high.

### 4.2. Specific Assumptions for Cost-Benefit Analysis

To illustrate the potential advantage of including the benefits from the aesthetic/amenity appreciation of the vegetated façades, we have undertaken simplified cost-benefit analyses of the two demonstration projects. The discount factor is set to 3% and the project horizon to 40 years [19], thus yielding an annuity factor for the project period equal to 23.81. Given a present value of single or a series of investment or benefits, we divide by the annuity factor to find amounts per year (that takes into account the impact of discounting). The CPI for EU27, from the European Central Bank, is: 93.58 for 2002, 111.91 for 2010, and 115.38 for 2011.

The benefits of the green walls comprise the noise attenuation for quieter façades/inner courtyards and other non-acoustic amenity benefits, e.g., the aesthetic effect. All the 48 apartments have one (quiet) side facing the inner courtyard, and will be affected by the noise attenuation due to the green wall at the entrance façade. It is assumed that noise impacts are omitted from the reviewed amenity valuation of green roofs/walls, such that we can simply add the aesthetic/amenity valuation to the dB-change valuation. At the outset it is assumed that the weighted averages of square metre non-acoustic unit values of green walls from the literature survey are applicable to the case project buildings considered in this paper. All apartments also benefit from the improved quality of the building block as such, having common access to the courtyard with improved acoustic and non-acoustic (visual) quality, as well as when passing into and out of the building complex. In addition, several of the dwellings will have the greenery of the façade in the direct view from inside the apartments. In the described demonstration studies not only the sound quality of the quiet side of the façades is improved, but also important parts of the neighbourhood soundscape such as in the courtyard of the building. The perceived benefit of this neighbourhood improvement will depend on other non-acoustic quality of the actual courtyards [55], in addition to the vegetated walls, and to what extent and how the courtyard areas are made use of by the residents.

Regarding project costs, these are additional costs compared to an alternative wall without greenery. The size of the façade improvements are of 58 m^2^ for the 3 m vegetated façade and 369 m^2^ for the 19.2 m façade. The wall is 9.6 m deep and the façades of both sides of the openings are covered. The cost of the vegetated façades is set to 500 €/m^2^, with a lifetime of 10 years. A lifetime of 50 (40–60) years is probably an appropriate estimate for (modern) green roofs [31]. Maintenance cost each year is 25 € per m^2^. These values are applicable for the project year 2011. This yields an annual cost (in 2011) of 56.91 € per m^2^ (including the investment cost and the annual maintenance).

Since there are substantial uncertainties associated with all of the estimated costs and benefits, a Monte Carlo simulation has been undertaken as a sensitivity analysis of the impact from varying the benefits of the aesthetic appreciation with ±50% and all other benefits and costs with ±30%. It is assumed that the number of beneficiaries vary ±15%. The uncertainties are input to the Monte Carlo simulations by drawing from a truncated normal distribution with standard deviation equal to the uncertainty multiplied with the estimate itself. For an effect of 4.5 dB(A) the bulk of the values lying between ±30% that is ±1.35 dB(A) has been assumed that results in a standard deviation of 1.35. The truncation of the normal distribution has no substantial impact, and is simply used as a mechanism to ensure that negative values or values very close to zero are not obtained.

## 5. Results

### 5.1. Meta-Analytic Weighted Averages

Table 2 summarises the weighing of the estimations for green roofs/walls. The weighted mean of these value estimates is only EUR 1.2 (per square metre green roof/wall per household per year, or EUR 0.5 per person, assuming a dweller or household size of 2.4). Yet, this result is nearly fully driven by the Kitakyushu estimate from Gao and Asami [42], which has a relative weight of almost 90%. As previously mentioned (see Section 3.2) the weighting scheme depends solely on the reported intrinsic quality of the studies and fails to take into account other sources of uncertainty. The outlier position suggests that here could be such differences that are unaccounted for in described in this paper weighing scheme. Removing this observation increases the weighted mean to EUR 5.8, which lies in the middle between the low outlier estimate, and that of a simple arithmetic mean (per person, the estimate is thus approximately 2.4 EUR, 2010 value.)

**Table 2:** Weighting of unit values, green roof/wall ^†^.

Study	Country Data	Uncertainty Measure	EUR-2010 Greenery Value	“Effect”, Unit Value (per m^2^)	W_g_ = 1/V_g_
Peck *et al.* (1999) [34]	Canada, Toronto	interval	1,044	20.88	0.048
Hunt (2008) [40]	Canada, Toronto	interval	895	17.90	0.027
Gao & Asami (2007) [42]	Japan, Tokyo	SE of parameter	87	3.46	0.368
	Japan, Kitakyushu	SE of parameter	17	0.69	6.351
Ichihara *et al.* (2010) [43]	US, New York	SE of parameter	859	17.18	0.014
Des Rosiers *et al.* (2002) [41]	Canada, Québec	SE of parameter	189	3.78	0.281
Tomalty & Komorowski (2010) [44]	Canada, Toronto	none	3,174	63.47	0.001
	Canada, Toronto	none	1,111	22.22	0.008

^†^ 1/*W_g_* yields *V_g_*, the variance, and the square root of *V_g_* yields the standard error estimate, *SE_g_*. See further explanations in Table 1.

### 5.2. The Acoustic and Non-Acoustic Benefits

Since the noise attenuation affects the quiet side, a scaling factor of 0.3 to adjust the benefits downwards has been used. Thus, from the nominal improvement of 4.5 dB(A) and 4.1 dB(A), the monetised noise valuation applies to, respectively 1.5 dB(A) and 1.37 dB(A). As a point of departure the HEATCO valuation of noise annoyance from 2002 have been used [19], yielding 10.095 € per person per dB(A) per year (valid for noise levels below 71 dB(A)). For the aesthetic appreciation 5.8 € per household converted to 2.4 €/person per year have been applied. These were entered as 2010 Euro values and subsequently updated by the CBA-tool to equivalent values for the project year, namely for the case of 2011 values. The number of persons in the demonstration buildings benefitting from the improvements (noise attenuation and aesthetics) in both cases equals approximately 115 (48 apartments with on the average 2.4 persons). One dwelling in the opposite building is assessed to benefit sufficiently from the direct view to the 3 m vegetated wall, while 6 apartments by the 19.2 m high vegetated wall can be also considered as aesthetic beneficiaries of the measure. Given the above, the annual benefit of the 4.5 dB(A) noise improvement gives 56.01 €/per person and 51.03 €/per person for the 19.2 metre wall. The annual aesthetic benefit in both cases is equal to 2.47 €/per person per m^2^ green wall (Table 3).

The benefit-cost ratios obtained from the calculations show that the sum of benefits is nearly four times as high as the costs. Also noteworthy is how amenity/aesthetic benefits are about 10 and 100 times higher than the benefits from the acoustic improvements. Given the tentative nature of the valuations and their application, the CBA for the situation where the low outlier value from the literature survey dominates the green wall unit value estimate, yielding 0.5 EUR per person (Table 4) can be also provided.

The lower green wall unit value assumption yields benefit-cost ratios just above unity. Moreover, the efficiency ranking has been also switched, with the low green wall ranked first, as the acoustic performance plays a relatively larger role and the lower wall (lower entrance opening) yields best noise attenuation.

**Table 3:** Calculation of benefit-cost (B/C) ratios, for the two demonstration projects with differently sized green walls—3 m high and 19.2 m high, assuming a green wall unit price of 2.4 EUR (2010) per m^2^ per person per year.

Measure	Size	Benefits	Costs	BC ratio
Green wall, 3 m façade opening	58 m^2^			
Investment costs			3,301	
Maintenance costs			1,450	
Noise attenuation benefits		1,932		
Amenity/aesthetic benefits		16,935		
Total		18,867	4,751	3.97
Green wall, 19.2 m façade opening	369 m^2^			
Investment costs			20,999	
Maintenance costs			9,225	
Noise attenuation benefits		1,761		
Amenity/aesthetic benefits		118,698		
Total		120,458	30,224	3.99

**Table 4:** Calculation of benefit-cost (B/C) ratios, for the two demonstration projects with differently sized green walls—3 m high and 19.2 m high, assuming a green wall unit price of 0.5 EUR (2010) per m^2^ per person per year.

Measure	Size	Benefits	Costs	BC ratio
Green wall, 3 m façade opening	58 m^2^			
Investment costs			3,301	
Maintenance costs			1,450	
Noise attenuation benefits		1,932		
Amenity/aesthetic benefits		4,234		
Total		6,166	4,751	1.30
Green wall, 19.2 m façade opening	369 m^2^			
Investment costs			20,999	
Maintenance costs			9,225	
Noise attenuation benefits		1,761		
Amenity/aesthetic benefits		29,674		
Total		31,435	30,224	1.04

### 5.3. Monte Carlo Simulations

**Figure 3:**
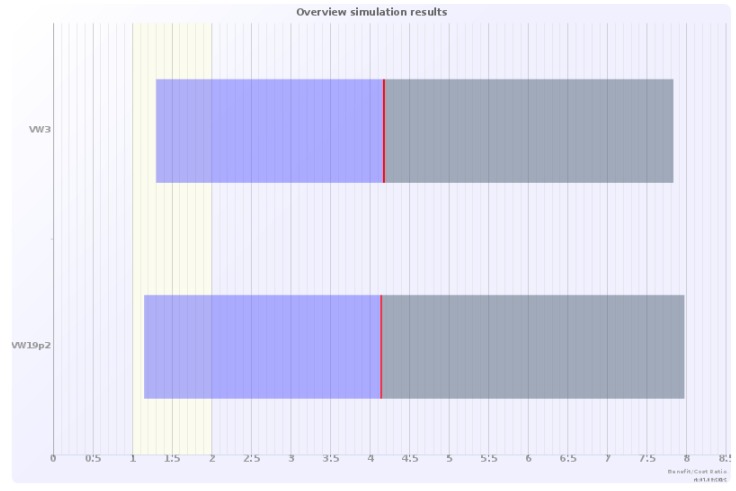
Monte Carlo simulations of benefit-cost (B/C) ratios, for the two demonstration projects with differently sized green walls—3 m high and 19.2 m high, assuming a green wall unit price of 2.4 EUR (2010) per m^2^ per person per year.

**Figure 4:**
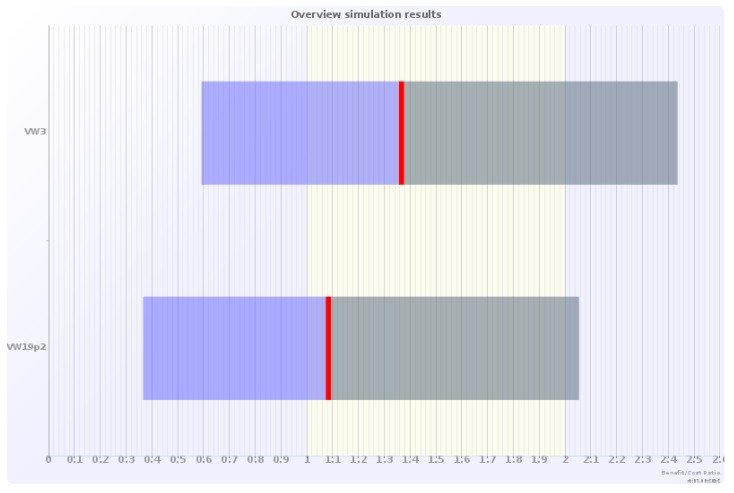
Monte Carlo simulations of benefit-cost (B/C) ratios, for the two demonstration projects with differently sized green walls—3 m high and 19.2 m high, assuming a green wall unit price of 0.5 EUR (2010) per m^2^ per person per year.

The sensitivity analysis of the CBA was accomplished by use of the Monte Carlo simulation (Figure 3 and Figure 4). For each CBA the benefit-cost ratio distribution was based on 10,000 simulations. The banded areas in Figure 3, representing the benefit-cost ratio simulation results, correspond to 90% of the simulated results. For the higher unit price estimate for aesthetic/amenity values, the benefit-cost ratio lies well above 1, in most cases above 2.

As expected, for the lower unit price estimate for aesthetic/amenity values, the simulated benefit-cost ratio for the green wall installations is considerably lower, around 1, indicating that economic efficiency will be questionable (Figure 4). Thus, the size of the unit value for green walls does have a clear impact on the estimated cost efficiency of these types of measures.

## 6. Discussion and Conclusions

The methodology and practice for economic assessment of noise-attenuation measures in quiet areas and vegetation-based noise attenuation measures have been incomplete. Valuation of noise attenuation has only been related to effects on the noisier façade of dwellings, not the effects on quiet areas, although soundscape improvements in quiet areas might be valued irrespective of an unchanged situation at the noisier façade [3,11,12,24,25,27,55]. Moreover, the economic assessment of noise-attenuation measures has neglected effects beyond the acoustic, which becomes particularly relevant for novel vegetation-based soundscape measures. This paper presented approaches to the economic valuation of noise attenuation in quiet areas and the inclusion of non-acoustic benefits; and by demonstration projects it has been shown that green walls can be economically promising as noise attenuation measures with additional amenity benefits.

Regarding the valuation of noise attenuation at the quiet façade, a slightly higher conversion factor than proposed by the CityHush project [12,27], that is, 30% instead of 20% has been applied. This has been motivated by the additional improvement of the courtyard soundscapes and the somewhat wider neighbourhood soundscape impacts of a project consisting of repeat interventions. An intervention rather than a steady state situation has been considered [56]. However, it is also possible to argue that the adverse noise environment along the street with heavy traffic reduces the perceived benefit of a quiet side improvement [22]. More detailed studies of how various aspects of the immediate soundscape of building complexes and neighbourhoods should subsequently be followed up by further psychological/economics research. Multi-site intervention studies, where contextual factors are allowed to vary, are needed.

There are basic methodological challenges in the application of hedonic pricing, as in other methods for valuation of environmental goods, e.g., that attributes like greenery and/or noise might be overestimated due to omission of other. A crude unit value estimates for urban greenery, namely square metre values for green roofs/walls has been proposed. These unit value estimates are simply based on averages of valuation estimates from the literature, predominantly hedonic pricing. The valuation studies have been carried out in various countries in the OECD area, with a majority having US origin. These studies covered different scopes of green roof/wall projects. In this paper, a simple transfer to Euro and an update to 2010-prices using standard CPI have been only adjustments of the original values. No additional adjustments or weightings using purchase price parities or the scope of the original valuation have been used. The weighted mean estimate from the surveys was 1.2 EUR per square metre per household per year, which increased to 5.8 EUR when the lowest estimate that had a 90% impact on the weighted mean has been eliminated. While the first of this estimate would yield benefit-cost ratios around unity for the two green wall demonstration projects that have been assessed, the second estimate would yield benefit-cost ratios around four. The relative economic value weight from aesthetics/amenities is in any case considerable, compared to noise attenuation values, but the exact unit value will of course affect economic efficiency estimates for a given project.

This study represents only a first step in economic assessment of vegetation-based noise attenuation measures, including a unit value for non-acoustic amenity benefits. The proposed simplistic approach can be regarded as applicable for a pilot CBA or a “mini-CBA” [57]. “For a mini-CBA one could apply known average values, both for effects and economic valuations, instead of going for the more elaborate estimation of case-specific effects and €. One of the limiting factors in the conduct of a full CBA evaluation is that the information on costs of implementing a measure often needs a full engineering assessment. This is time-consuming and costly. As part of the mini-CBA it can be possible to work with approximate data on the cost of measures.” ([58] p. 26, 82) The current alternative to the green wall CBA, including benefit estimates of noise attenuation in a quiet area and simplistic economic unit values of amenity/aesthetic impacts, is a CBA where these effects are assigned zero economic value. Whereas one should be careful not to draw crystal clear implications from CBA of the two demonstration projects for green walls, there is an indication of a very important lesson: noise control measures that jointly and simultaneously produce multiple benefits, say noise reduction and enhancement of aesthetical/ambient qualities, might be considerably more economically efficient than noise control measures that do not provide such additional benefits. It may seem obvious that measures producing various benefits jointly (and simultaneously) are preferable [23]. However, one might find many examples from reality and economic studies where only one effect or a limited set of effects is assessed, as if other effects didn’t exist. Omitting welfare-affecting effects from economic analyses yield partial studies that might twist resulting estimates, e.g., estimated net benefits, or benefit-cost ratios, from cost-benefit analyses [59]. Broadening the perspective to include aesthetics and other benefits, in addition to the benefits in sound quality, can potentially generate an economic rationale for deploying such measures. If results obtained in this study hold up in subsequent studies, measures such as vegetated walls should be included in national or regional noise policies and be seriously considered by municipal and other authorities intending to improve urban soundscapes. An advantage of the study is also that future research that can relate their findings to the valuation proposed in this paper, and develop the value assessment further. 
